# Low dose of chlorine exposure exacerbates nasal and pulmonary allergic inflammation in mice

**DOI:** 10.1038/s41598-018-30851-6

**Published:** 2018-08-22

**Authors:** Isabella Santos de Genaro, Francine Maria de Almeida, Deborah Camargo Hizume-Kunzler, Henrique Takachi Moriya, Ronaldo Aparecido Silva, João Carlos Gonçalves Cruz, Renan Boeira Lopes, Renato Fraga Righetti, Rodolfo de Paula Vieira, Mitiko Saiki, Milton Arruda Martins, Iolanda de Fátima Lopes Calvo Tibério, Fernanda Magalhães Arantes-Costa, Beatriz Mangueira Saraiva-Romanholo

**Affiliations:** 1 Public Employee of Sao Paulo Hospital (IAMSPE), Sao Paulo, Brazil; 2 Department of Medicine (LIM 20), School of Medicine, University of Sao Paulo, Sao Paulo, Brazil; 3 Department of Physical Therapy (LaPEx), State University of Santa Catarina, Florianopolis, Brazil; 4 Biomedical Engineering Laboratory, Escola Politecnica, University of Sao Paulo, Sao Paulo, Brazil; 5 University City of Sao Paulo (UNICID), Sao Paulo, Brazil; 6 Sírio-Libanês Hospital, Sao Paulo, Brazil; 7 Universidade Brasil, Post-graduation Program in Bioengenering, São Paulo, Brazil and Brazilian Institute of Teaching and Research in Pulmonary and Exercise Immunology (IBEPIPE), São José dos Campos, Brazil; 8 Nuclear and Energy Research Institute, IPEN-CNEN/SP, Sao Paulo, Brazil

## Abstract

Work-exacerbated asthma (WEA) is defined as preexisting asthma that worsens with exposure to irritants [e.g., chlorine (Cl_2_) derivatives] in the workplace. The maximum allowable concentration in the workplace of Cl_2_ exposure is 3 mg/ m^3^ (described in OSHA). We investigated in an experimental asthma model in mice the effects of a single exposure to a sodium hypochlorite dose with this allowed chlorine concentration and a tenfold higher dose. Acute chlorine exposure at 3.3 mg/m^3^ in the OVA-sensitized group increased eosinophils in the peribronquial infiltrate, cytokine production, nasal mucus production and the number of iNOS positive cells in the distal lung compared to only sensitized mice. The exposure to a higher dose of 33.3 mg/m^3^ in the OVA-sensitized group resulted in an increase in respiratory system elastance, in the total and differential numbers of inflammatory cells in bronchoalveolar lavage fluid, IL-4, IL-5, and IL-17 in the lungs, eosinophils in peribronquial infiltrate and mucus content in nasal compared to non-exposed and sensitized animals. In this asthma model, chorine exposures at an allowable dose, contributed to the potentiation of Th2 responses. The functional alterations were associated with increased iNOS and ROCK-2 activation in the distal lung.

## Introduction

Allergic respiratory conditions are among the most prevalent disorders in Western populations^1,2^. The exact cause of the increase in the prevalence of these conditions remains unclear^1,2^.

Because of its wide inner surface, the respiratory system is usually exposed to particles and gases, and it is also easily exposed to allergens and irritant compounds^3,4^. The airway epithelium works as a barrier that regulates water and ion transport and, through mucociliary clearance, transports inhaled particles. Furthermore, the epithelium participates in inflammation, immune defense against pathogens, tissue remodeling, and cytokine production^4,5^. This complex role allows epithelial cells to communicate with mesenchymal cells in the airway and transfer signals to other cells, such as immune cells, which are capable of producing inflammatory mediators and amplifying the injury process^4^.

Once exposed to inhaled substances, such as air pollutants, allergens or pathogens, epithelial damage and cell disruption occur^4^, and the airway afferent nerve promotes the release of mediators and stimulates changes in the respiratory response^6^. Some of these mediators are the inducible nitric oxide (iNOS) enzyme^7^ and Rho-kinase^8^. Changes in ROCK-2, which modulates contraction, affect smooth muscle and alter the actin cytoskeleton, cell adhesion, motility, migration and contraction^8–10^.

The response to an allergen is primarily mediated by the interaction between dendritic cells (DCs) and lymphocytes, whereas irritant agents might cross the cell membrane and react with local proteins, creating or revealing new epitopes^11^ and generating oxygen free radicals^12^, ultimately resulting in respiratory injury^13,14^. This injury can increase epithelial permeability, allowing access by allergens to the subepithelial DCs^15^, thus becoming an efficient adjuvant to present these proteins to macrophages and DCs, releasing a wide range of inflammatory cytokines^16^.

Animal studies have also shown that exposure to respiratory irritants can increase pulmonary responsiveness^17^ and activate immune cells, thus contributing to the release of inflammatory cytokines (e.g., IL-4 and IL-17) in the lungs and the production of reactive oxygen (ROS) and reactive nitrogen species (RNS)^18–20^. This status might also contribute to DNA damage^18,21–23^.

Among environmental factors, exposure to disinfectants based on chlorine products has been identified as a source of irritation and airway inflammation^24^. Products that contain Cl_2_ derivatives, such as sodium hypochlorite, are widely used by cleaning workers and are directly linked to respiratory illness^25–28^.

These situations are defined as work-related asthma (WRA), which represents a health problem with significant potential for acute morbidity, long-term disability, and severe social and economic impacts^29,30^_._

Occupational asthma (OA) is the most common form of WRA and accounts for approximately 15% of all adult-onset asthma^31,32^. OA has emerged as a relevant focus of investigations related to asthma physiopathology^31^. The Cl_2_ time-weighted average exposure to be considered occupational exposure is 0.5 ppm over 8 h or 1 ppm over 10 minutes^33^. The Occupational Safety and Health Administration (OSHA) recommends that the allowable permissible exposure level (PEL) for chlorine is 1 ppm or 3 mg/m^3^ ^34^.

However, the mechanisms that mediate the induction or facilitation of allergic conditions in WRA remain unclear. A growing number of animal studies have been performed, but the results remain controversial^35–37^. Hox *et al*. demonstrated that chronic nasal instillation of NaClO with 3 ppm of active Cl_2_ before ovalbumin (OVA) sensitization resulted in airway hyperresponsiveness (AHR) without affecting sensitization to the antigen^36^. In contrast, Kim *et al*., who also used an experimental OVA model, found that exposure to chronic low doses of chlorine through a vaporized 5% NaClO solution aggravated allergen-induced airway inflammation^37^.

To our knowledge, no previous study has addressed whether acute exposure to chlorine in a background of pre-existing allergic lung inflammation could affect the distinctive features of asthma. Thus, we first assessed, in naïve animals and afterwards in an asthma model, the effects of a single exposure to a sodium hypochlorite dose with the maximum allowable concentration and a tenfold higher dose described in the OSHA to assess lung function, inflammation, remodeling, the oxidative stress pathway and ROCK-2 expression.

## Results

### Phase I: Chlorine gas exposure in naïve animals and its effects on pulmonary responsiveness and lung inflammation

#### Acute chlorine exposure increases airway bronchoconstriction and inflammatory cells in naive mice

**Figure 1:**
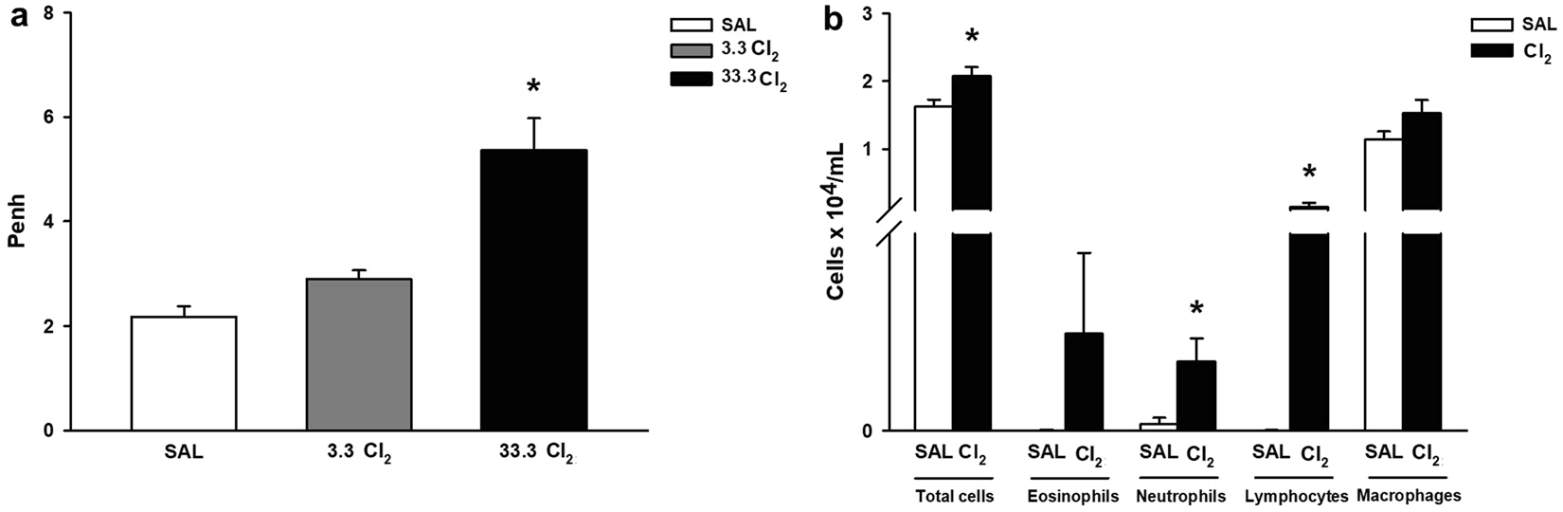
Enhanced pause (Penh) and inflammatory cells in health mice. These values are expressed as the mean ± SE (n = 6). **(a)** Penh: *significantly different compared to the others groups (*p* < 0.001). **(b)** Inflammatory cells in the BALF: *significantly different when total cells (*p* = 0.024), neutrophils (*p* = 0.026) and lymphocytes (*p* = 0.031) in the Cl_2_ exposed animals were compared to the SAL group.

In the first phase of this experiment, we evaluated chlorine gas exposure by inhalation of increasing concentrations of sodium hypochlorite. We analyzed the enhanced pause (Penh) to the dose-response curve (Fig. 1a) of each group and observed an increase in lung responsiveness when mice were exposed to 33.3 Cl_2_ compared to the SAL dose (*p* < 0.001) and compared to the 3.3 Cl_2_ dose (*p* < 0.001).

When inflammatory cells (Fig. 1b) were analyzed, we observed an increase in total cells (*p* = 0.024), neutrophils (*p* = 0.026) and lymphocytes (*p* = 0.031) in Cl_2_-exposed animals compared to the SAL group.

### Phase II: Chlorine gas exposure in a model of allergic pulmonary inflammation

#### Acute chlorine exposure exacerbates respiratory system responsiveness

**Figure 2:**
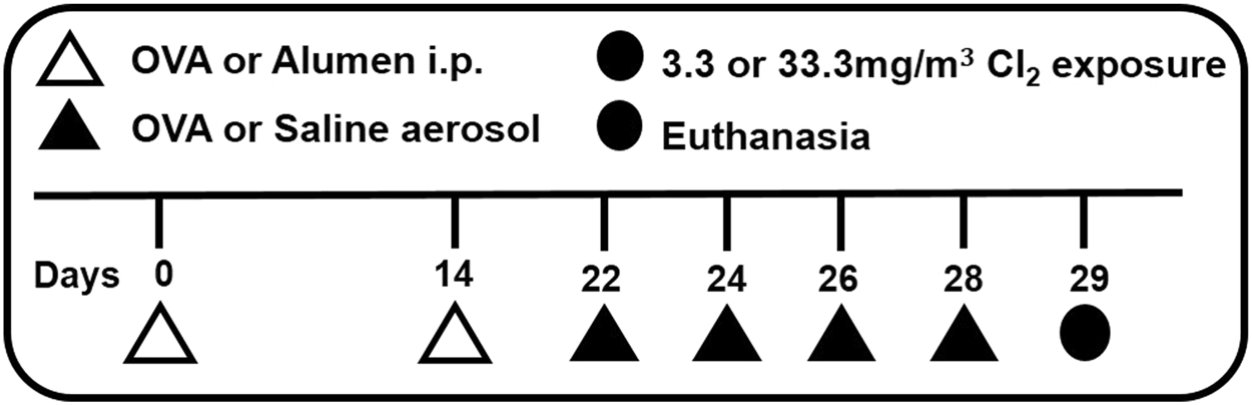
Timeline of the experimental protocol. On days 0 and 14 mice of OVA, OVA +3.3 Cl_2_, OVA + 33.3 Cl_2_ received intraperitoneal (I.P.) injections of OVA solution with vehicle, while the SAL group received only vehicle (open triangles). Aerosol challenges with a 1% OVA solution were performed four times on days 22, 24, 26, and 28 (closed triangles) and animals in the SAL group received only aerosolized saline. A single Cl_2_ exposure was performed on day 29 (closed circle). After Cl_2_ exposure, lung mechanics were measured, and the animals were euthanized.

**Figure 3:**
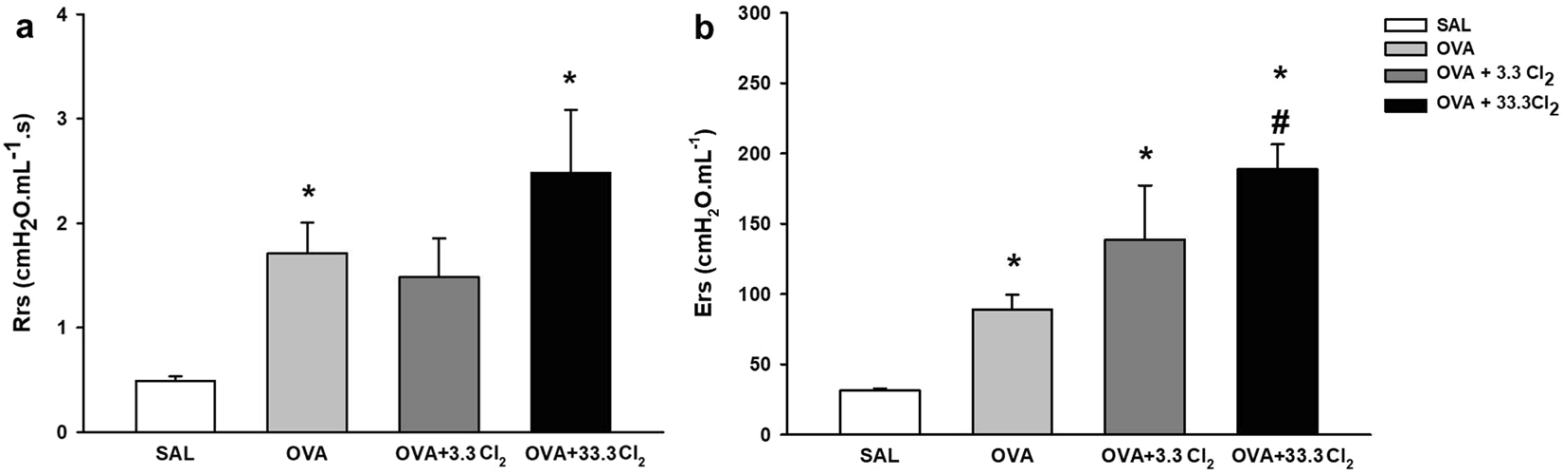
Resistance (Rrs) and elastance (Ers) of the respiratory system. These values are expressed as the mean ± SE (n = 6). **(a)** Rrs: *significantly different compared to the Saline group (*p* < 0.009 for OVA group and *p* < 0.001 for OVA + 33.3Cl_2_). **(b)** Ers *significantly different compared to the Saline group (*p* = 0.013 for OVA group and *p* < 0.001 for both Cl_2_ exposed groups) and ^#^significantly different compared to the OVA group (*p* < 0.001).

Compared to the SAL group (Fig. 2), respiratory system resistance (Rrs) (Fig. 3a) and elastance (Ers) (Fig. 3b) were exacerbated in OVA-sensitized animals (*p* = 0.009 and *p* = 0.013, respectively).

Furthermore, compared to OVA-sensitized animals, only the OVA + 33.3 Cl_2_-exposed animals exhibited an increase in Ers (*p* < 0.001).

#### Acute exposure to the higher dose of chlorine increases pulmonary inflammation

**Figure 4:**
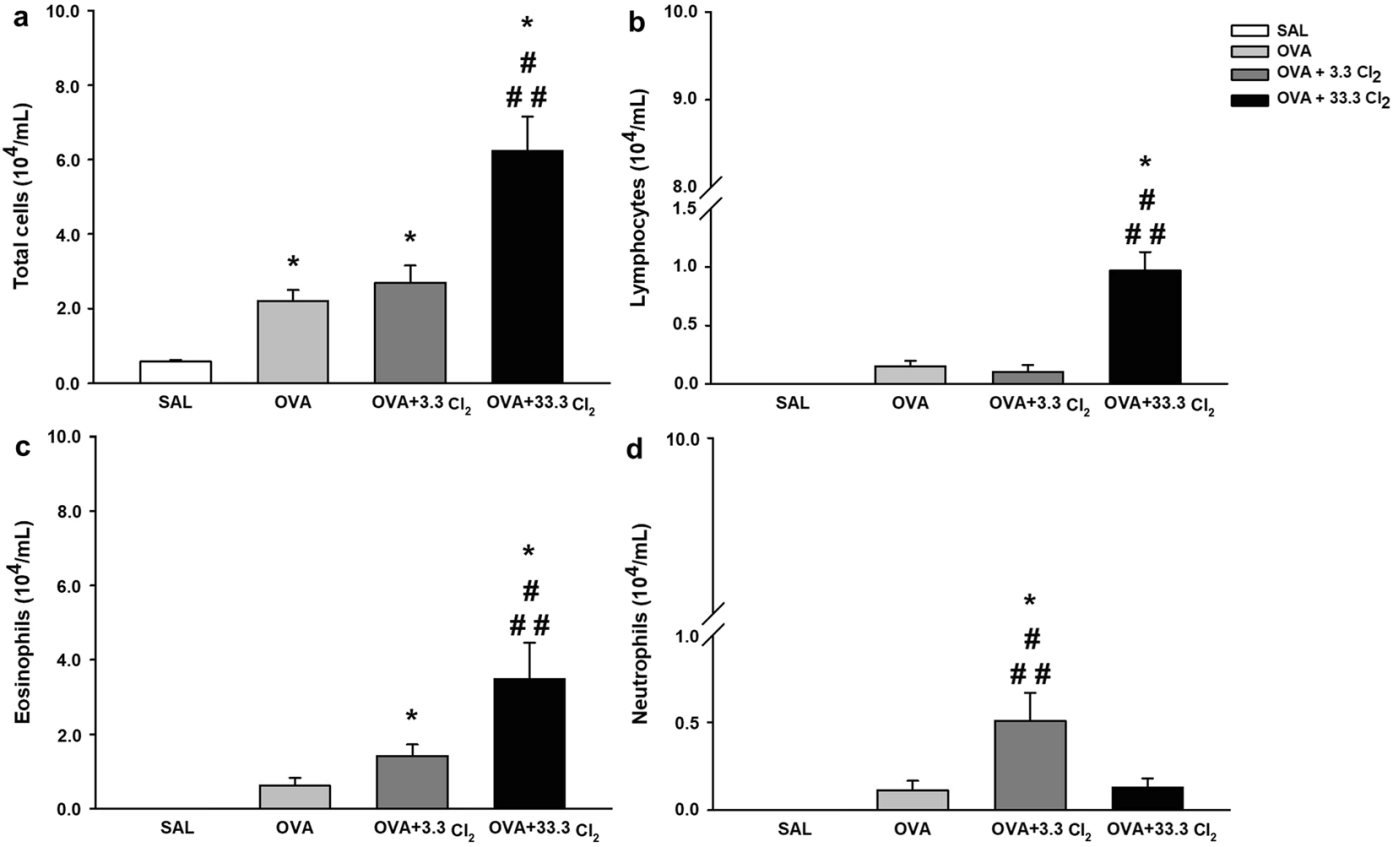
Counting of inflammatory cells in the BALF. The values are expressed as the mean ± SE (n = 6). **(a)** Total cells: *significantly different compared to the Saline group (*p* = 0.037 for OVA group, *p* = 0.011 for OVA + 3.3 Cl_2_ group and *p* < 0.001 for OVA + 33.3 Cl_2_ group), ^#^significantly different compared to the OVA group (*p* < 0.001) and ^##^significantly different compared to the OVA + 3.3 Cl_2_ group (*p* < 0.001). **(b)** Lymphocytes: *significantly different compared to the Saline group (*p* < 0.001), ^#^significantly different compared to the OVA group (*p* < 0.001) and ^##^significantly different compared to the OVA + 3.3 Cl_2_ exposed group (*p* < 0.001). **(c)** Eosinophils: *significantly different compared to the Saline group (*p* < 0.001), ^#^significantly different compared to the OVA group (*p* < 0.001) and ^##^significantly different compared to the OVA + 3.3 Cl_2_ exposed group (*p* < 0.006). **(d)** Neutrophils: *significantly different compared to the Saline group (*p* < 0.001), ^#^significantly different compared to the OVA group (*p* = 0.005) and ^##^significantly different compared to the OVA + 33.3 Cl_2_ exposed group (*p* = 0.009).

Figure 4 shows the effects of acute sodium hypochlorite exposure on the number of leukocytes in the BALF.

Compared to the SAL group, OVA-sensitized animals exhibited an increase in the number of total cells (Fig. 4a) when not exposed to Cl_2_ (*p* = 0.037). Furthermore, animals in the OVA + 33.3 Cl_2_ group showed a higher number of inflammatory cells compared to the OVA and OVA + 3.3 Cl_2_ groups (*p* < 0.001).

Figure 4b shows that exposure to a higher dose of chlorine in OVA-sensitized animals resulted in an increased number of lymphocytes compared to the OVA and OVA + 3.3 Cl_2_ groups (*p* < 0.001).

Exposure to a higher dose of chlorine resulted in an increased number of eosinophils (Fig. 4c) compared to the OVA (*p* < 0.001) and OVA + 3.3 Cl_2_ (*p* < 0.006) groups.

Furthermore, Fig. 4d shows that exposure to the maximal allowable dose of chlorine in OVA-sensitized animals resulted in an increased number of neutrophils compared to the OVA and OVA + 33.3 Cl_2_ groups (*p* = 0.005 and *p* = 0.009, respectively).

When macrophages were analyzed, no differences between groups were observed. The number of macrophages in each experimental group was as follows (mean ± SE): SAL group (0.16 ± 0.11 × 10^4^/mL), OVA group (1.32 ± 0.27 × 10^4^/mL), OVA + 3.3 Cl_2_ (1.50 ± 0.66 × 10^4^/mL) and OVA + 33.3 Cl_2_ group (1.65 ± 0.37 × 10^4^/mL).

#### iNOS expression is only increased by chlorine gas in the distal lung but not in the peribronchial infiltrate

**Figure 5:**
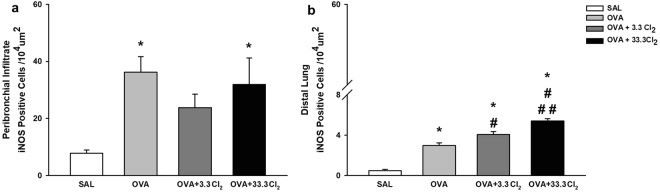
iNOS positive cells. The values are expressed as the mean ± SE (n = 6). **(a)** Positive cells in the airways: *significantly different compared to Saline group (*p* < 0.005 for OVA group and *p* < 0.014 for OVA + 33.3Cl_2_). **(b)** Positive cells in the lung parenchyma: *significantly different compared to Saline group (*p* < 0.001 for all groups), ^#^significantly different compared to the OVA group (*p* = 0.008 and *p* < 0.001, respectively) and ^##^significantly different compared to the OVA + 3.3 Cl_2_ group (*p* = 0.001).

As shown in Fig. 5a, quantification of the number of iNOS-positive cells in the peribronchial infiltrate was increased only in OVA-sensitized animals not exposed to Cl_2_ (*p* < 0.005) compared to the SAL group.

On the other hand, analyses in the distal lung (Fig. 5b) revealed an increase in the number of iNOS-positive cells in the OVA-sensitized group compared to the SAL group (*p* < 0.001) and an increase in both groups exposed to Cl_2_ (*p* = 0.008 and *p* < 0.001, respectively). Furthermore, animals exposed to the high dose of chlorine differed compared to the group that received the maximum allowable concentration of chlorine (*p* = 0.001).

#### Acute chlorine exposure to a high dose of Cl_2_ increased ROCK-2-positive cells in the distal lung but not in the peribronchial infiltrate

**Figure 6:**
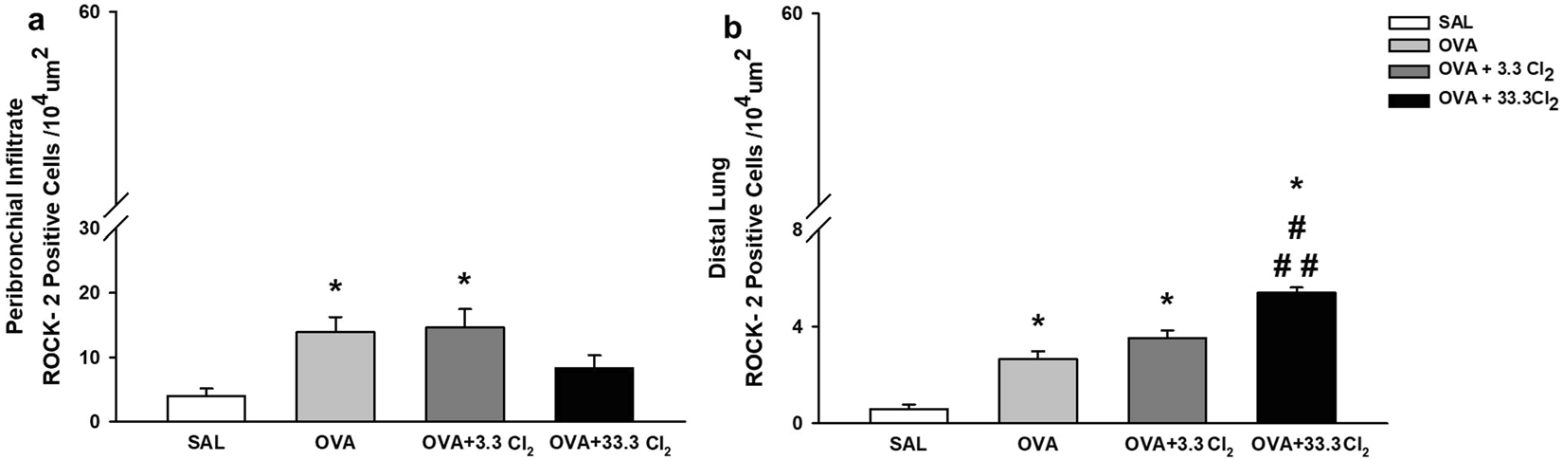
ROCK-2 positive cells. The values are expressed as the mean ± SE (n = 6). **(a)** Positive cells in the airways: *significantly different compared to Saline group (*p* = 0.007). **(b)** Positive cells in the distal lung: *significantly different compared to Saline group (*p* < 0.001 for all groups), ^#^significantly different compared to the OVA group (*p* < 0.001) and ^##^significantly different compared to the OVA + 3.3 Cl_2_ group (*p* < 0.001).

The cellular expression of ROCK-2 in the peribronchial infiltrate is shown in Fig. 6a. We observed increased ROCK-2 expression in OVA-sensitized animals not exposed to Cl_2_ (*p* < 0.007) compared to the SAL group.

In the lung parenchyma (Fig. 6b), this increase was observed in the OVA-sensitized group compared to the SAL group (*p* < 0.001) and in OVA + 33.3 Cl_2_ animals compared to the OVA group (*p* < 0.001) and the group that received the maximum allowable concentration of chlorine (*p* < 0.001).

#### Acute chlorine exposure increases the number of eosinophils in the peribronchial infiltrate

**Figure 7:**
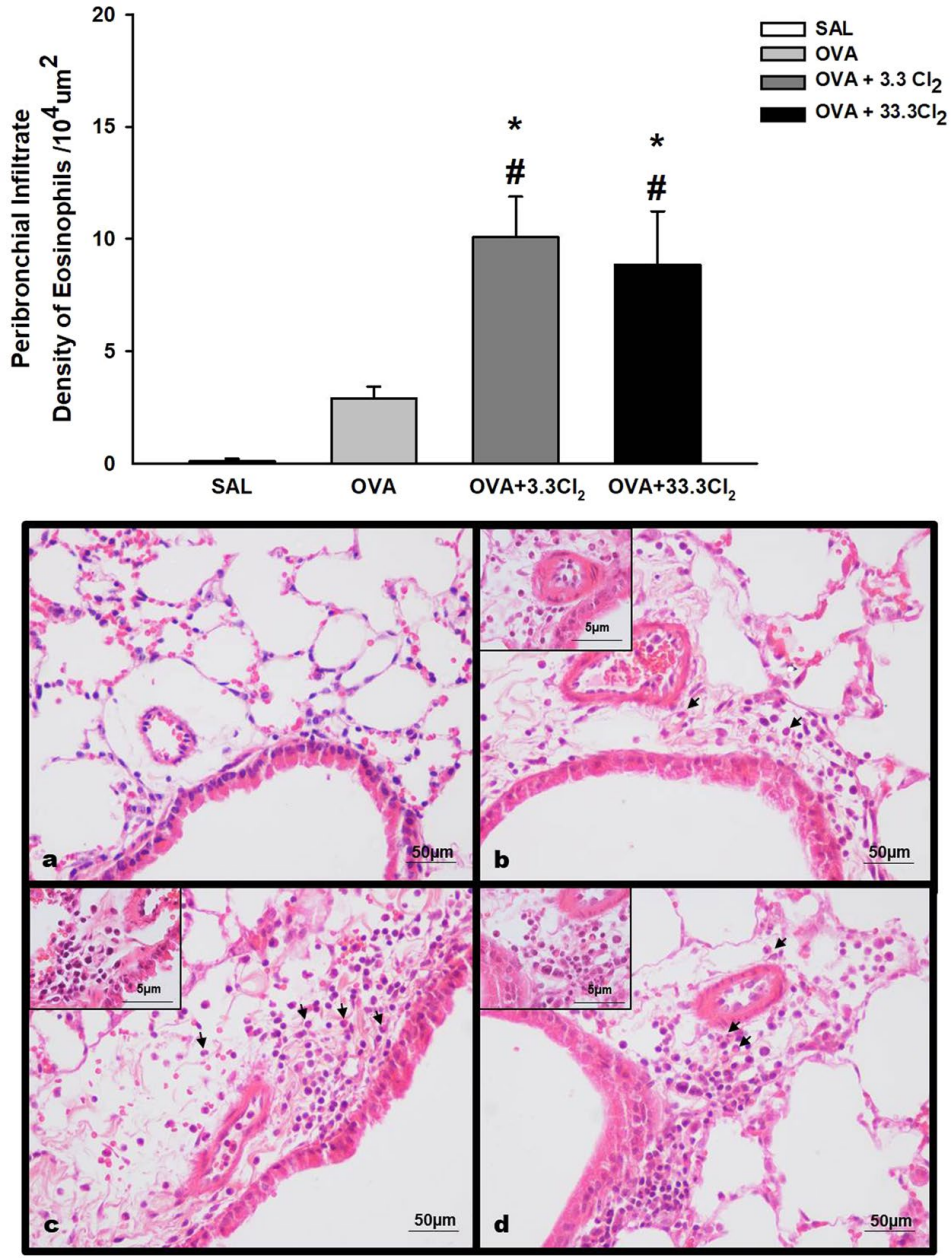
Eosinophils cells in the peribronchial space. The values are expressed as the mean ± SE (n = 6). *significantly different compared to Saline group (*p* < 0.001 for both Cl_2_ groups). ^#^Significantly different compared to the OVA group (*p* = 0.002 for OVA + 3.3 Cl_2_ and *p* = 0.008 for OVA + 33.3 Cl_2_). Photomicrography panel of peribronchial infiltrate. All figures are presented at a magnification of 400×, scale bars = 50 µm with an insert at a magnification of 1000×, scale bar = 5 µm. **(a)** SAL group, **(b)** OVA group, **(c)** OVA + 3.3 Cl_2_ and **(d)** OVA + 33.3 Cl_2_.

Figure 7 shows the effects of Cl_2_ exposure on the number of eosinophils in the peribronchial infiltrate. Acute chlorine exposure at both concentrations tested significantly increased the number of eosinophils in OVA-sensitized animals compared to the OVA group (*p* = 0.002 and *p* = 0.008, respectively).

#### Acute chlorine exposure did not increase the number of neutrophils in the peribronchial infiltrate

To exclude the possibility that cells observed in the peribronchial infiltrate were neutrophils, we performed an immunohistochemical analysis. The cell count of the experimental groups was as follows (mean ± SE): SAL group (0.75 ± 0.31 cells/10^4^ µm^2^), OVA group (0.34 ± 0.14 cells/10^4^ µm^2^), OVA + 3.3 Cl_2_ (1.14 ± 0.33 cells/10^4^ µm^2^) and OVA + 33.3 Cl_2_ (0.78 ± 0.30 cells/10^4^ µm^2^). No significant differences between experimental groups were observed.

#### Acute chlorine exposure did not enlarge the mean linear intercept (Lm)

The Lm was analyzed to evaluate lung tissue destruction; no differences between groups were observed. The Lm of each experimental group was as follows (mean ± SE): SAL group (27.35 ± 2.16 µm), OVA group (30.52 ± 0.99 µm), OVA + 3.3 Cl_2_ (30.66 ± 1.27 µm) and OVA + 33.3 Cl_2_ (32.92 ± 1.76 µm).

#### Interstitial edema is not increased by chlorine exposure

Interstitial edema, which was evaluated by score, was low in all experimental groups and did not present differences between groups. The results are as follows (mean ± SE): SAL group (4.11 ± 0.81), OVA group (5.27 ± 0.89), OVA + 3.3 Cl_2_ (6.38 ± 0.45) and OVA + 33.3 Cl_2_ (3.77 ± 0.65).

#### Acute chlorine exposure increases lung cytokine levels

**Figure 8:**
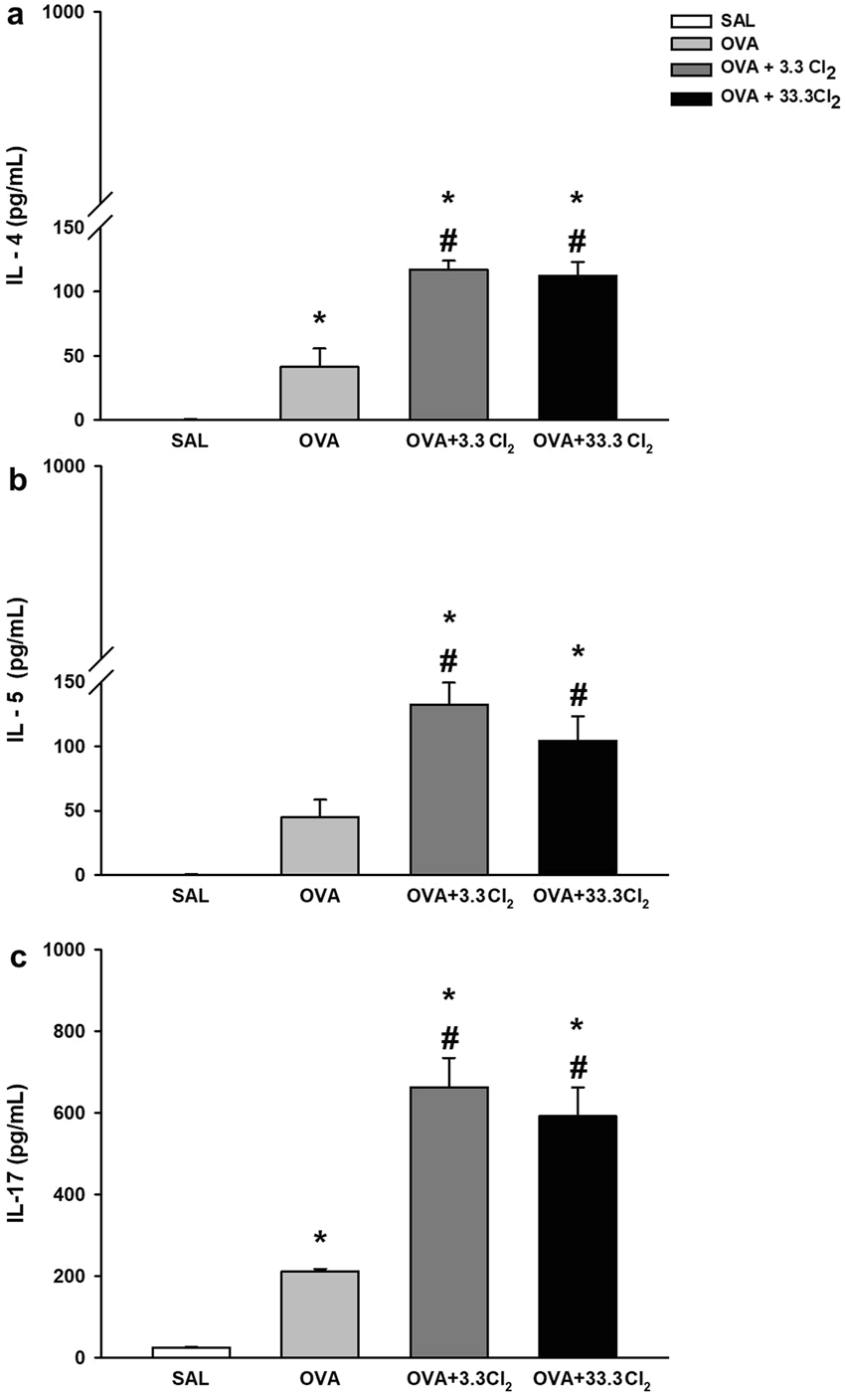
Cytokine levels in lung homogenates. The values are expressed as the mean ± SE (n = 6). **(a)** IL-4 levels: *significantly different compared to Saline group (*p* = 0.007 for OVA group and *p* < 0.001 for both Cl_2_ groups). ^#^Significantly different compared to the OVA group (*p* < 0.001). **(b)** IL-5 levels: *significantly different compared to the Saline group (*p* < 0.001 for both Cl_2_ groups). ^#^Significantly different compared to OVA group (p = 0.001). **(c)** IL-17 levels: *significantly different compared to the Saline group (*p* = 0.035 for OVA group and *p* < 0.001 for both Cl_2_ groups). ^#^Significantly different compared to the OVA group (*p* < 0.001).

By analyzing lung tissue homogenates, we found that chlorine exposure at both concentrations tested significantly increased the lung levels of IL-4, IL-5, and IL-17 in all groups (Fig. 8a–c, respectively).

The OVA group not exposed to Cl_2_ exhibited increased IL-4 levels compared to the SAL group (*p* = 0.007), and the OVA groups exposed to both concentrations showed an increase compared to OVA non-hypochlorite-exposed mice (*p* < 0.001).

Similarly, IL-5 lung levels showed a significant increase in OVA mice exposed to 3.3 or 33.3 Cl_2_ compared to the OVA group not exposed (*p* < 0.018 and *p* < 0.001, respectivily). Additionally, both concentrations tested increased the IL-17 levels relative to the OVA group not exposed (*p* < 0.001), and the OVA-sensitized group had a higher level of pulmonary IL-17 compared to the SAL group (*p* = 0.035).

#### Acute chlorine exposure increases the acid mucus content in the nasal epithelium

**Figure 9:**
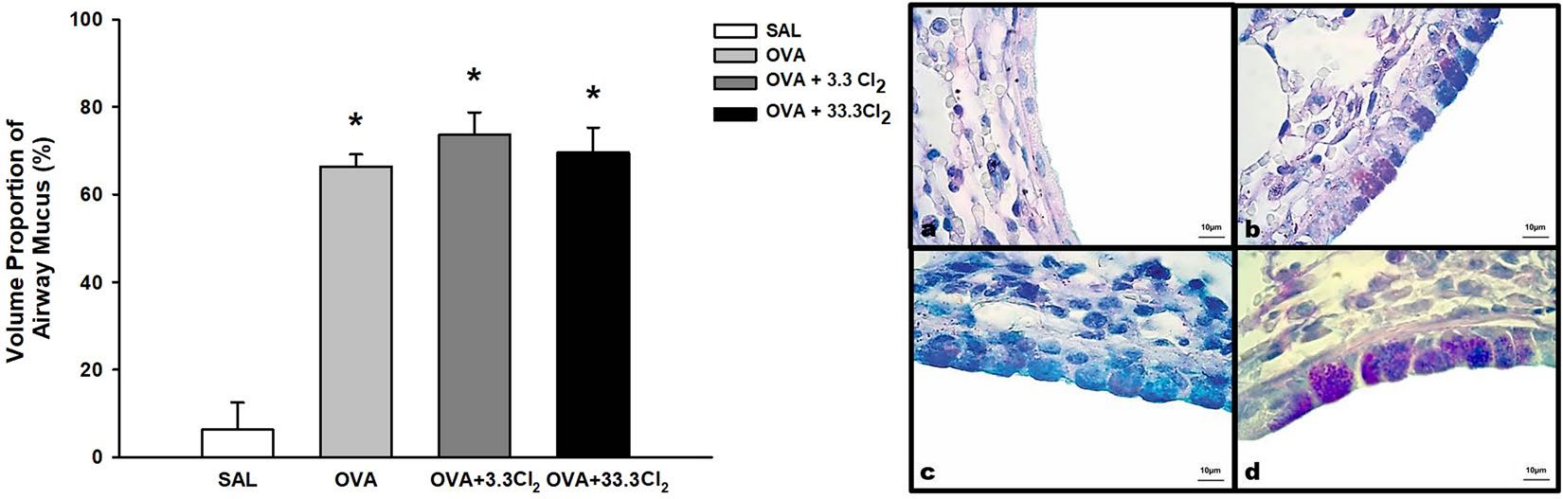
Volume proportion of airway mucus. The values are expressed as the mean ± SE (n = 6). *Significantly different compared to the Saline group (*p* < 0.001 for all groups). Photomicrography panel of airway epithelium. All figures are presented at a magnification of 1000×, scale bars = 10 µm. **(a)** SAL group, **(b)** OVA group, **(c)** OVA + 3.3 Cl_2_ and **(d)** OVA + 33.3 Cl_2_.

In this experimental model, we observed that the OVA group not exposed to Cl_2_ was significantly different compared to the SAL group (*p* < 0.001). We measured both neutral (data not shown) and acid mucus substances, but the analysis of neutral mucus *per se* did not show a significant difference between groups (*p* > 0.05). However, when neutral and acid mucus were analyzed together, we observed an increase in total mucus content (Fig. 9).

**Figure 10:**
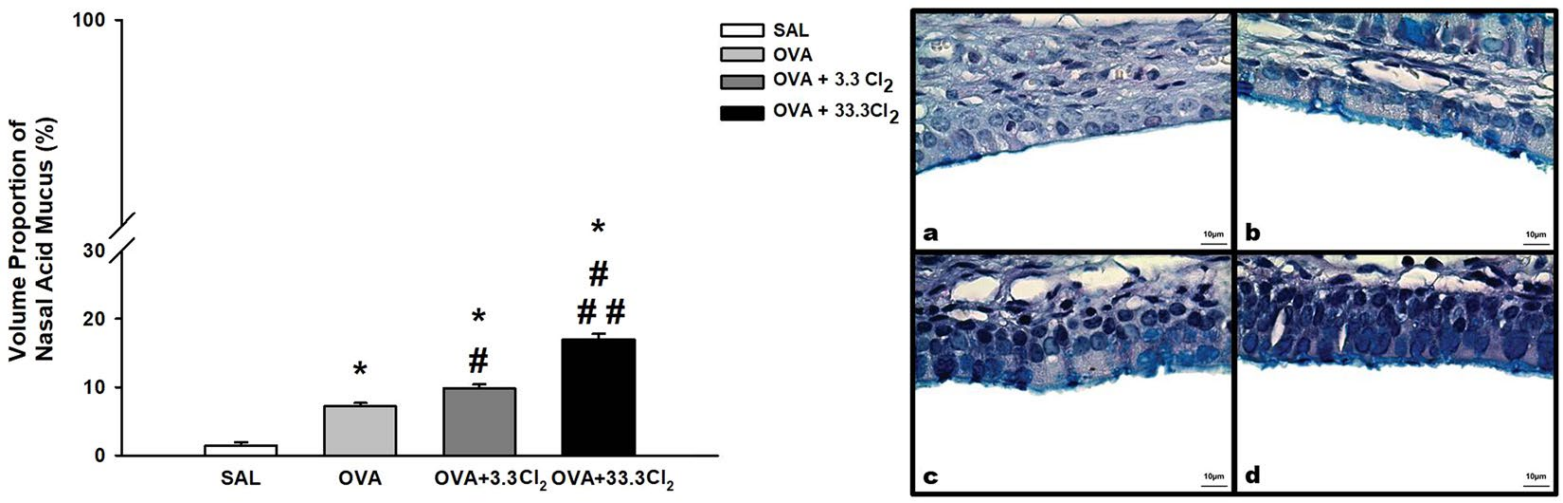
Volume proportion of nasal acid mucus. The values are expressed as the mean ± SE (n = 6). *Significantly different compared to the Saline group (*p* < 0.001 for all groups). ^#^Significantly different compared to the OVA group (*p* = 0.015 for OVA + 3.3 Cl_2_ and *p* < 0.001 for OVA + 33.3 Cl_2_) ^##^significantly different compared to the OVA + 3.3 Cl_2_ group (*p* < 0.001). Photomicrography panel of nasal epithelium. All figures are presented at a magnification of 1000×, scale bars = 10 µm. **(a)** SAL group, **(b)** OVA group, **(c)** OVA + 3.3 Cl_2_ and **(d)** OVA + 33.3 Cl_2_.

We also observed an increase in acid mucus in the nasal epithelium (Fig. 10) in the OVA-sensitized group compared to the SAL group (*p* < 0.001). Furthermore, both groups exposed to Cl_2_ exhibited an increase in acid mucus compared to the OVA group (*p* = 0.015 and *p* < 0.001, respectively), and animals exposed to the higher dose differed compared to animals that received the maximum allowable concentration of chlorine (*p* < 0.001).

## Discussion

This study was performed to provide experimental evidence related to a common work situation referred to as WEA, which is defined as current asthma that worsens in response to various work-related factors, such as aeroallergens, changes in temperature, exercise, and exposure to irritants^29,30,38–45^. Among the environmental irritant factors, exposure to chlorine derivatives, especially disinfection products, has been highlighted^29,30^.

Our results demonstrated, for the first time, that acute chlorine exposure at 3.3 mg/m^3^ in the OVA-sensitized group increased eosinophils in the peribronchial space, cytokine production, nasal mucus production and iNOS-positive cells in the distal lung compared to only sensitized mice. Exposure to a higher dose (33.3 mg/m^3^ Cl_2_) in the OVA-sensitized group resulted in an increase in respiratory system elastance, total and differential numbers of inflammatory cells in the BALF, IL-4, IL-5, and IL-17 in the lungs, eosinophils in the peribronchial space, mucus content in the nasal epithelium and the number of iNOS- and ROCK-2-positive cells in the distal lung compared to non-exposed and sensitized animals. Furthermore, naïve mice exposed to increased doses of chlorine showed alterations in respiratory mechanics and a higher number of neutrophils and lymphocytes in the BALF.

AHR is usually employed to investigate the functional status in many pulmonary conditions, including those involving toxic irritants and allergens^17,36,46^. In this study, we showed that OVA-sensitized and OVA-challenged mice acutely exposed to chlorine at concentrations of 33.3 mg/m^3^ (tenfold higher than the maximum allowable concentration of chlorine in the workplace) exhibited exacerbated elastance of the respiratory system.

Our results agree, in part, with those reported by Hox *et al*., who observed a slight increase in the resistance of the respiratory system in OVA-sensitized mice and those chronically exposed to nasal instillations of NaClO at 3 ppm^36^ and by Kim *et al*.^37^, who observed airway hyperresponsiveness in sensitized animals and challenged mice exposed to naturally vaporized 5% sodium hypochlorite during four weeks. In our experimental model, we found increased elastance of the respiratory system in OVA-sensitized mice acutely exposed to the higher dose of Cl_2_ and in the Penh of naïve mice exposed to increasing doses of chlorine.

In addition to hyperresponsiveness and transient bronchospasm, chlorine gas inhalation can lead to temporary mucous membrane irritation and pulmonary edema due to an increase in vascular permeability, which allows an influx of eosinophils, neutrophils and lymphocytes into the lungs, causing enhanced pulmonary inflammation^1,15,47^. We observed a higher influx of eosinophils in the peribronchovascular space in animals acutely exposed to chlorine at both concentrations tested. This inflammation may be related to the enhancement of the lung mechanical response in our experiment.

Additionally, the influx of inflammatory cells into the injury site promotes the release of inflammatory cytokines^11,48,49^. In experimental asthma models and clinical studies of asthmatic populations, increases in pro-inflammatory cytokines, such as IL-4 and IL-5, are directly related to the differentiation, proliferation, recruitment, and survival of inflammatory cells in allergic inflammation^50–53^.

In our experimental model, 24 h after the last challenge, the OVA groups exposed to both chlorine concentrations showed bronchial eosinophilia and bronchial Th2 cytokine production, as previously published^17,46,49,50^. This eosinophilia was confirmed by immunohistochemical analysis using an antibody specific for neutrophils, and no differences between experimental groups were observed, suggesting that the majority of these cells were eosinophils.

Interestingly, acute Cl_2_ application had an additional major impact on the allergic response, as evaluated by measuring inflammatory cells in the BALF, with an increase in the number of total cells, neutrophils, eosinophils and lymphocytes, and by measuring IL-4, IL-5, and IL-17 levels in homogenized lung tissue.

Curiously, some studies have also shown that other inflammatory factors may be activated more directly by irritating substances through the activation of pro-inflammatory signaling pathways within cells, as occurs in the bronchial epithelium^54–56^. The iNOS enzyme synthesizes L-arginine to produce nitric oxide (NO) in response to pro-inflammatory mediators, including cytokines, in chronic diseases such as asthma^57,58^. High levels of chlorine (100 ppm) have already been described as a mechanism to induce iNOS production by recruiting neutrophils^14,59^.

Our results are consistent with this idea, where OVA-sensitized animals exhibited an increase in oxidative stress markers following iNOS production in the lungs^60^. However, OVA-sensitized and chlorine-exposed animals did not show an increase in iNOS-positive cells in the airways, which can be explained by the chlorine dose used in this study. On the other hand, the number of positive cells in the lung parenchyma were increased in the OVA group and in both OVA groups exposed to chlorine.

Another signaling pathway that may increase inflammatory cytokines, the infiltration of inflammatory cells into airways and changes in the respiratory response is the Ro-kinase pathway. Righetti *et al*.^8^ demonstrated that the inhibition of Ro-kinase reduced distal lung tissue responsiveness and the release of inflammatory mediators. Although the inflammatory response in the BALF showed an increase in the number of inflammatory cells compared to the low dose of chlorine, cytokine analyses and the number of eosinophils in the peribronchial infiltrate did not differ between chlorine groups. Because the Ers value is dependent on the distal airway and distal lung hyperresponsiveness, we believed it was important to evaluate oxidative stress, which is associated with the Rho-kinase system^8^.

In our experimental study, we observed an increase in the number of ROCK-2-positive cells in the airways and in the lung parenchyma of OVA-sensitized animals. Furthermore, animals exposed to an acute high dose of Cl_2_ exhibited an increase in ROCK-2 expression in the lung parenchyma. Changes in respiratory elastance were accompanied with an increase in ROCK-2 expression, suggesting the influence of this mediator in the pulmonary response.

The airways are normally protected with a thin layer of mucus, a hydrogel that protects the epithelium from harmful particles, pathogens and chemicals^61^. They are transported out of the lungs by this gel formed with water and mucins (glycoproteins)^62^.

In some diseases, acidification of the mucus alters its properties, increasing its viscosity and impairing mucociliary clearance^63,64^. Because of this increased viscosity, the acid mucus is normally present in the nasal epithelium, acting as a barrier against pathogens and allergens. Daily, the nasal epithelium produces 1.5 to 2 L of mucus that is renewed approximately every 20 minutes. By contrast, the particles reach the airway lining in 15 minutes to 2 h after inhalation, and the amount of mucus secreted each day is approximately 10 mL^61^.

The Th2 cytokines, such as IL-4, that were increased in the present study may stimulate mucin production^61^. We believe that the systemic effects of Th2 cytokines that were potentialized by chlorine exposure contributed to the results observed in the nasal mucus. Therefore, this evidence contributes to a better understanding of the differences between the nasal and airway mucus results.

Here, we show that single chlorine exposure induced an increase in mucus content and the nasal epithelium in the OVA groups. These results may be partially explained by the oxidative modification of free functional groups of proteins and the induction of lipid peroxidation, which are caused by chlorine and result in increased vascular permeability, the release of inflammatory cytokines, the influx of inflammatory cells, and increased mucus production^11,48,65^.

A limited number of studies have reported the quantitative exposure assessment to chlorine in the workplace^66,67^. Since most respiratory toxicity appears to be related to chlorine released from a sodium hypochlorite solution, we determined the chlorine levels once this could be related to the PEL of 1 ppm or 3 mg/m^3^ provided by OSHA^34^ and a tenfold higher dose. Even at allowable chlorine concentrations, we observed an increase in inflammatory cells in the airway walls, increased cytokine production, and increased nasal mucus production when allergic inflammation was combined with acute exposure to irritant products.

We are also aware of the limitations of the animal models of asthma, including some differences in the immune responses^53^. However, the OVA model used in our study is a well-established model of allergic asthma that we have used to study another acute condition^46,50,53,56,68–70^. Furthermore, more experimental and clinical research addressing acute or chronic chlorine provided from sodium hypochlorite exposure should be performed to understand the signaling pathways, remodeling and oxidative stress in an allergic or health condition.

On the other hand, no previous work has been performed using acute exposure to the accepted levels of chlorine in a model of asthma that has reported increased inflammation even without changes in pulmonary function, suggesting the importance of the evaluation of inflammation in asthmatic patients without changes in pulmonary function, as well as a review of the permitted level of chlorine exposure in this group of people.

In conclusion, this experimental asthma model revealed that chorine exposure, at an allowable dose, contributed to the potentiation of Th2 responses. Furthermore, the functional alterations were associated with increased iNOS and ROCK-2 activation in the distal lung.

## Materials and Methods

The present study was submitted and approved by the Review Board for Human and Animal Studies of the School of Medicine of the University of Sao Paulo (n° 029-10). Male BALB/c mice (25–30 g, 6 weeks old, specific pathogen-free [SPF]) were obtained from the Animal Facility of the School of Medicine of the University of Sao Paulo. Animals were maintained in controlled conditions of temperature (22 ± 2 °C), humidity (70–75%), and dark/light cycle (12 h; lights on at 06:00 am) and allowed food and water *ad libitum*. All animal care and experimental procedures followed the Guide for the Care and Use of Laboratory Animals^71^_._

### Phase I: Chlorine gas exposure in naïve animals and its effects on pulmonary responsiveness and lung inflammation

#### Animals and experimental groups

Twelve male BALB/c mice were divided into two groups as follows (n = 6 mice/group): (1) naïve mice exposed to saline (SAL); (2) naïve mice exposed to increasing concentrations of chlorine, 3.3 and 33.3 mg/m^3^ (Cl_2_).

#### Whole-body plethysmography

In the first phase of experiments, we determined the minimal dose of chlorine to be used in the second phase protocol. Animals were sensitized to ovalbumin by placing naive mice in a chamber and evaluating the pulmonary mechanics to increasing doses of sodium hypochlorite (0.00%, 0.03% [3.3 mg/m^3^] and 0.3% [33.3 mg/m^3^]) using a whole-body plethysmography system (BUXCO, Winchester, UK). Briefly, data acquisition was performed using a transducer connected to a computer system that continuously measured the box pressure–time wave, which calculated the Penh, which is related to air bronchoconstriction^3,72^.

After measuring the baseline Penh, animals received aerosolized saline followed by two sodium hypochlorite concentrations (0.03% and 0.3%) through an inlet in the chamber for 3 minutes. The Penh values were collected for 5 minutes and averaged. Next, animals were anesthetized (thiopental sodium, 33 mg/kg i.p.) and tracheotomized for bronchoalveolar lavage fluid collection, and the numbers of total and differential cells were counted.

#### Total and differential cell counts in the BALF

Three instillations of 0.5 mL of 0.9% NaCl were performed via a tracheal cannula, and the lungs were gently washed. All washes were combined, processed, and analyzed. The total cell number was counted in a Neubauer hemocytometer chamber, and 300 cells (macrophages, lymphocytes, eosinophils and neutrophils) were counted in each Diff Quick-stained cytospin slide in a blinded fashion.

#### Chlorine level determination

To determine the chlorine levels in the exposure atmosphere, samples of filter paper (55-mm diameter, Whatman 1, Sigma-Aldrich Brazil Ltda., Sao Paulo, Brazil) were nebulized with NaClO. The chlorine level was determined by neutron activation analysis. Filter paper samples were folded and placed in polyethylene envelopes for irradiation in the IEA-R1 Nuclear Research Reactor of Nuclear and Energy Research Institute along with the synthetic standard of chlorine.

The chlorine mass in the synthetic standard was 500 µg, and the irradiation time was 25 s under a thermal neutron flux of 1.9 × 10^12^ n cm^−2^ s^−1^. After a decay time of 5 minutes, gamma ray activities were measured using a hyperpure Ge detector coupled to a Digital Spectrum Analyzer DSA 1000 (Canberra, Meriden, CT, USA). The gamma spectrum was processed using Genie 2000 software version 3.1 (Canberra). Chlorine was identified by the ^38^Cl peak with a half-life of 37.24 minutes and a gamma ray energy of 1642.69 keV. The chlorine mass was calculated by the comparative method^73^ after discounting chlorine present in the filter paper (blank value).

After the procedures, the chlorine masses per filter paper area were 3.3 (an accepted level of chlorine) and 33.3 mg/m^3^ (10-times higher), respectively.

### Phase II: Chlorine exposure in a model of allergic pulmonary inflammation

#### Animals and experimental groups

Twenty-four male BALB/c mice were divided into four groups as follows (n = 6 mice/group): (1) mice that were non-sensitized and not exposed to chlorine (SAL); (2) mice that were OVA-sensitized and OVA-challenged but not exposed to chlorine (OVA); (3) mice that were OVA-sensitized and OVA-challenged and exposed to 3.3 mg/m^3^ of chlorine (OVA + 3.3 Cl_2_); and (4) mice that were OVA-sensitized and OVA-challenged and exposed to 33.3 mg/m^3^ of chlorine (OVA + 33.3 Cl_2_).

#### OVA model of allergic pulmonary inflammation

BALB/c mice were sensitized with an intraperitoneal (i.p.) injection of 50 µg of OVA with aluminum hydroxide (vehicle) on days 0 and 14, while the SAL group received vehicle only. Mice were exposed to aerosolized OVA (1%) or saline (0.9% NaCl) for 30 minutes on days 22, 24, 26 and 28 (Fig. 2)^17,56^.

On day 29, animals were anesthetized (thiopental sodium, 33 mg/kg i.p.), tracheotomized, and placed in a plethysmograph chamber connected to a small animal ventilator (Harvard Apparatus, South Natick, MA, USA).

#### Respiratory system mechanical assessment

Differential pressure transducers (Honeywell, Freeport, IL, USA) were applied to measure the whole-body plethysmography pressure (from which changes in lung volume were derived) and tracheal pressure. Volume and tracheal pressure signals were acquired at 200 Hz over 10 s with a 12-bit analog-to-digital converter (DT 01-EZ, Data Translation, Marlboro, MA, USA) and stored (LABDAT, RHT-InfoData, Inc., Montreal, QC, Canada) in a microcomputer. Airflow changes were obtained via electronic derivation of the volume signal. Rrs and Ers were obtained by applying the single compartment model of the respiratory system to the experimental data (pressure, volume, and flow signals). All data were analyzed using computer software for the assessment of respiratory mechanics (ANADAT 4.0, RHT-InfoData, Inc., Montreal, QC, Canada).

Animals were anesthetized and tracheotomized and connected to the ventilator. Next, baseline Ers and Rrs values were collected, and chlorine gas exposure was performed by nebulization of 0.03% (3.3 mg/m3) and 0.3% (33.3 mg/m^3^) NaClO solutions (Sigma Chemical Co., St. Louis, MO, USA). An ultrasonic device (Respira Max, NS, LTDA., Sao Paulo, Brazil) nebulized either aerosolized saline or NaClO solutions through the air inlet of the ventilator for 2 minutes. The respiratory mechanics data were assessed after 30 s and after 1, 2, and 3 minutes. The total duration of lung mechanics evaluation was approximately 12 minutes for each animal, and the results are expressed as the maximum responses of Rrs and Ers. Animals in the SAL and OVA groups received aerosolized saline only.

After these procedures, the animals were euthanized by rapid exsanguination of the abdominal aorta. The bronchoalveolar fluid (BALF) was then collected, and lungs were excised.

#### Total and differential cell counts in the bronchoalveolar lavage fluid (BALF)

Same as in phase I.

### Evaluation of iNOS, ROCK-2 and neutrophils positive cells in the peribronchial infiltrate

Immunohistochemistry for iNOS, ROCK-2 and neutrophil detection was performed using a protocol modified from Martins-Oliveira *et al*.^74^. Lung sections (5-µm-thick) were deparaffinized and hydrated. Antigen retrieval was performed, and sections were washed in phosphate-buffered saline (PBS) and blocked with 3% hydrogen peroxide at room temperature. Next, sections were incubated with rabbit antibody anti-iNOS (cod. RB-9242-P; LabVision, Neo-Markers, Fremont, CA, USA) at a dilution of 1:300, goat anti-ROCK-2 (cod. sc-1851; Santa Cruz Biotechnology, Santa Cruz, CA, USA) at a dilution of 1:50 and rabbit anti-neutrophil elastase (cod. ab21595, Abcam, Cambridge, MA, USA) at a dilution of 1:1500. All primary antibodies were diluted in bovine serum albumin (BSA) overnight (16–18 h) in a humid chamber at 4–8 °C. Subsequently, sections were washed in PBS and incubated with a secondary antibody (Vector ABCElite, horseradish peroxidase [HRP]; Vector Laboratories, Burlingame, CA, USA, cods. PK-6101 anti-rabbit and PK-6105 anti-goat) at 37 °C in a humidified chamber. Three additional 5-minutes washes in PBS were performed and samples were revealed with 3,3-diaminobenzidine (DAB) (cod. K3468, Dako Citomation, Fort Collins, CO, USA) for 5 minutes. Subsequently, tissues were washed with tap water and counterstained with Harris hematoxylin^74^. Cell density was assessed as the number of cells divided by the respective edema area (10^4^ cells/µm^2^) in five peribronchovascular structures. The analysis was performed by an optical microscope provided with an integrating eyepiece containing a known area (10^4^ μm^2^ at a magnification of 1000×) of 50 lines and 100 points^75^.

### Evaluation of eosinophils in the peribronchial infiltrate

Sections (5-µm-thick) were stained with hematoxylin and eosin (H&E) and used to measure the edema area (data not shown) and eosinophil density; counts were performed at a magnification of 1000× in the peribronchovascular space using the point-counting technique. Cell density was assessed as described above^75^.

### Evaluation of iNOS- and ROCK-2-positive cells in the distal lung

Using the point-counting technique, we quantified the number of iNOS- and ROCK-2-positive cells divided by the number of points hitting the alveolar septa wall area in each field; 10–15 random non-overlapping fields were analyzed, and the results are expressed as 10^4^ cells/area^8^.

### Mean linear intercept (Lm)

The Lm was measured using a microscope with an integrating eyepiece containing a known area (50 lines and 100 points) at 200× magnification and was calculated as the number of times the reticular lines intercepted the alveolar walls in the distal lung parenchyma. For each animal, 20 non-overlapping fields were randomly analyzed. Values are expressed in micrometers. Sections (5-µm-thick) were stained with H&E^56,76^.

### Morphometric analysis for interstitial edema (IE) evaluation

We used a weighted scoring system (scale 0 to 4) to quantify interstitial edema, where 0 represents no visible evidence and 4 represents complete involvement of each field. Briefly, by using a light microscope, the extent of each scored characteristic per field of view (score 0–4) was determined by four non-overlapping fields of view at 100× (0–16), 200× (0–16) and 400× (0–16) magnification^77^. Final score for each animal was determined as an average of the score at the 3 magnifications (IE = score at 100×+ score at 200×+ score at 400×/3) ranging from 0 to 16.

### Measurement of cytokines in the lung homogenate

After removal, the right lung was homogenized and centrifuged at 900 × g for 7 minutes at 4 °C (Powerlyzer, Mo Bio Laboratories, Carlsbad, CA, USA), and the supernatant was stored at −70 °C until subsequent analysis. The levels of IL-4, IL-5 and IL-17 in the lung homogenate were measured using ELISA Duo Set Kits (R&D Systems, Minneapolis, MN, USA) according to the manufacturer’s instructions.

### Measurement of lung mucus

After formalin fixation, the left lung was embedded in paraffin, and 5-µm-thick sections were stained with Schiff Periodic Acid and Alcian Blue (PAS-AB) at a pH of 2.5^78^. An optical microscope with an integrating eyepiece (Weibel reticle)^75^ containing a known area (10^4^ μm^2^ at a magnification of 1000×) of 50 lines and 100 points was used to measure the total (neutral and acidic) mucus content and the total airway epithelial area of non-cartilaginous airways (data not shown). The volume proportions of total mucus were obtained by dividing the total number of points hitting the positive mucus area by the number of total points hitting the airway epithelial area^17^. Morphometric measures were performed in five airways per animal according to previous studies^17^. The airways were chosen randomly according to optical scanning of each slide. All morphometric measurements were performed in a blinded fashion.

### Measurement of nasal epithelium acid mucus

The nasal cavity was flushed through the nasopharyngeal orifice with 5 mL of 10% neutral-buffered formalin in a retrograde manner, and the head was fixed in formalin for 24 h and then decalcified in 5% ethylenediamine tetraacetic acid (EDTA) for two weeks. Next, 5-μm-thick sections were taken immediately from the nasal cavity from the posterior region to the upper incisor teeth^79^ and stained with PAS-AB^78^. The region of the nasal respiratory epithelium that was analyzed included the epithelium lining the nasal septum. Ten randomly selected fields from each slide were studied, and the number of points corresponding to the total area of the epithelium in each field was calculated. The acidic mucous substance was quantified as described above^75,80^.

### Statistical analysis

Comparisons of the BALF from the first phase were performed using a t-test. One-way analysis of variance followed by the Holm-Sidak test was performed for other analyses for multiple comparisons^17^.

The significance level was adjusted to 5% (*p* < 0.05). Sigma Stat 3.5 software (San Jose, CA, USA) was used for all statistical analyses. All values are expressed as the mean ± standard error (SE).

## Data Availability

All data generated or analyzed during this study are included in this published article.
