# Author Correction: Low dose of chlorine exposure exacerbates nasal and pulmonary allergic inflammation in mice

**DOI:** 10.1038/s41598-018-36667-8

**Published:** 2018-12-12

**Authors:** Isabella Santos de Genaro, Francine Maria de Almeida, Deborah Camargo Hizume-Kunzler, Henrique Takachi Moriya, Ronaldo Aparecido Silva, João Carlos Gonçalves Cruz, Renan Boeira Lopes, Renato Fraga Righetti, Rodolfo de Paula Vieira, Mitiko Saiki, Milton Arruda Martins, Iolanda de Fátima Lopes Calvo Tibério, Fernanda Magalhães Arantes-Costa, Beatriz Mangueira Saraiva-Romanholo

**Affiliations:** 1 Public Employee of Sao Paulo Hospital (IAMSPE), Sao Paulo, Brazil; 2 Department of Medicine (LIM 20), School of Medicine, University of Sao Paulo, Sao Paulo, Brazil; 3 Department of Physical Therapy (LaPEx), State University of Santa Catarina, Florianopolis, Brazil; 4 Biomedical Engineering Laboratory, Escola Politecnica, University of Sao Paulo, Sao Paulo, Brazil; 5 University City of Sao Paulo (UNICID), Sao Paulo, Brazil; 6 Sírio-Libanês Hospital, Sao Paulo, Brazil; 7 Universidade Brasil, Post-graduation Program in Bioengenering, São Paulo, Brazil and Brazilian Institute of Teaching and Research in Pulmonary and Exercise Immunology (IBEPIPE), São José dos Campos, Brazil; 8 Nuclear and Energy Research Institute, IPEN-CNEN/SP, Sao Paulo, Brazil

Correction to: *Scientific Reports* 10.1038/s41598-018-30851-6, published online 22 August 2018

The Acknowledgements section in this Article is incomplete.

“This work was supported by Sao Paulo Research Foundation, (FAPESP) grant 2009/53904-9 and grant 2012/15165-2, Experimental Therapeutic Laboratory I (LIM 20), School of Medicine, University of Sao Paulo, School of Medicine Foundation (FFM/USP) and Financial Assistance to Educational Project or Research (AUXPE/PROAP) grant 2696/2013.”

should read:

“This work was supported by Sao Paulo Research Foundation, (FAPESP) grants 2009/53904-9, 2012/15165-2 and 2018/20380-6, Experimental Therapeutic Laboratory I (LIM 20), School of Medicine, University of Sao Paulo, School of Medicine Foundation (FFM/USP) and Financial Assistance to Educational Project or Research (AUXPE/PROAP) grant 2696/2013.”

